# Sensing Cytosolic DNA Lowers Blood Pressure by Direct cGAMP-Dependent PKGI Activation

**DOI:** 10.1161/CIRCULATIONAHA.123.065547

**Published:** 2023-08-07

**Authors:** Jie Su, Pierre Coleman, Angeliki Ntorla, Rhys Anderson, Michael J. Shattock, Joseph R. Burgoyne

**Affiliations:** 1 School of Cardiovascular and Metabolic Medicine & Sciences, King’s College London; The British Heart Foundation Centre of Excellence, The Rayne Institute, St Thomas’ Hospital, United Kingdom.

**Keywords:** blood pressure, cGAS protein, mouse, cyclic GMP-dependent protein kinases, cyclic guanosine monophosphate-adenosine monophosphate, sepsis

## Abstract

**BACKGROUND:**

The major cytosolic DNA sensor cyclic GMP-AMP synthase (cGAS) has emerged as a key mediator of inflammation that underlies cardiovascular disease. On interaction with double-stranded DNA, cGAS generates the second messenger 2′,3′-cyclic GMP-AMP (cGAMP) that directly binds to and activates the stimulator of interferon genes, which in turn leads to enhanced expression of genes encoding interferons and proinflammatory cytokines. Here, we show that cGAMP generated by cGAS also directly activates PKGI (cGMP-dependent protein kinase 1), a mechanism that underlies crosstalk between inflammation and blood pressure regulation.

**METHODS:**

The ability of cGAS and cGAMP to activate PKGI was assessed using molecular, cellular, and biochemical analyses, and in myography experiments, as well. The release of cGAMP from the endothelium was measured using an ELISA, and its uptake into the vascular smooth muscle was assessed using molecular and biochemical approaches, including the identification and targeting of specific cGAMP transporters. The blood pressure of wild-type and cGAS^–/–^ mice was assessed using implanted telemetry probes. cGAS was activated by in vivo transfection with G3-YSD or mice were made septic by administration of lipopolysaccharide.

**RESULTS:**

The detection of cytosolic DNA by cGAS within the vascular endothelium leads to formation of cGAMP that was found to be actively extruded by MRP1 (multidrug resistance protein 1). Once exported, this cGAMP is then imported into neighboring vascular smooth muscle cells through the volume-regulated anion channel, where it can directly activate PKGI. The activation of PKGI by cGAMP mediates vasorelaxation that is dependent on the activity of MRP1 and volume-regulated anion channel, but independent of the canonical nitric oxide pathway. This mechanism of PKGI activation mediates lowering of blood pressure and contributes to hypotension and tissue hypoperfusion during sepsis.

**CONCLUSIONS:**

The activation of PKGI by cGAMP enables the coupling of blood pressure to cytosolic DNA sensing by cGAS, which plays a key role during sepsis by mediating hypotension and tissue hypoperfusion.

**Clinical Perspective**

**What Is New?**

- Cyclic GMP-AMP (cGAMP) synthase (cGAS) can mediate lowering of blood pressure through direct PKGI (cGAMP-dependent protein kinase I) activation.
- cGAS within the vascular endothelium generates cGAMP that is actively extruded by MRP1 (multidrug resistance protein 1) and then imported into neighboring vascular smooth muscle cells through the volume-regulated anion channel.
- The activation of PKGI by cGAS/cGAMP plays a key role during sepsis by mediating hypotension and tissue hypoperfusion.

**What Are the Clinical Implications?**

- Our findings provide novel insight into the pathological processes that underlie sepsis.
- Targeting processes that mediate the formation or transport of cGAMP may provide new therapeutics to treat sepsis.

**Editorial, see p 1035**

The cyclic GMP-AMP (cGAMP) synthase (cGAS) responds to pathogenic DNA derived from bacteria and viruses, and cytosolic self-DNA, as well.^1,2^ Once activated on DNA binding, cGAS generates the second messenger cGAMP that, in turn, binds and activates the stimulator of interferon genes (STING), leading to the induction of genes associated with the innate immune response.^3–5^ The activation of this pathway plays an important role in cardiovascular disease and has been implicated in myocardial ischemic injury,^6^ the development of heart failure,^7^ cardiac dysfunction during sepsis,^8^ and diabetic cardiomyopathy.^9^ In addition, the activation of cGAS-STING has also been found to play an important role in the development of sporadic aortic aneurysm and dissection.^10^

Here, we report that PKGI (cGMP-dependent protein kinase 1) can be directly activated by cGAMP. This has important implications because PKGI plays a crucial role in the cardiovascular system by regulating blood pressure, platelet aggregation, cardiomyocyte proteostasis, cardioprotection, and cardiac contractility.^11–13^ Consistent with the central role of PKGI in regulating blood pressure, its activation by cGAS/cGAMP limited the constriction of isolated vessels and led to hypotension in telemetered mice.^14^ This process was also dependent on the endothelium, where cGAMP generated by cGAS is exported through MRP1 (multidrug resistance protein 1) and then taken up into vascular smooth muscle cells through the volume-regulated anion channel (VRAC), where it can then activate PKGI. This novel mechanism was found to underlie the inflammatory changes in blood pressure mediated by cGAS, which caused hypotension and tissue hypoperfusion during sepsis.

## METHODS

The data supporting the findings of this study are available from the corresponding author on reasonable request.

### Isolation of Rat Aortic Smooth Muscle Cells

Dissected rat thoracic aorta was cleaned of surrounding fat and connective tissue, opened lengthways, and cut into 1- to 2-mm small sections. Aortic sections were placed lumen side down in tissue culture plates and covered with media (DMEM supplemented with 10% FCS and 1% penicillin/streptomycin) to encourage vascular smooth muscle cell (VSMC) outgrowth onto the plate surface. After ≈5 days, when the VSMCs had grown and adhered to the plate, the aortic sections were removed, with cells then maintained in culture media until confluent.

### Cell Culture

All cells were maintained at 37 °C in a 5% CO_2_ humidified incubator and were regularly passaged. Rat aortic smooth muscle cells were passaged every 2 to 3 days when confluency reached >80% and cultured in DMEM (Gibco, catalog no. 31966021) supplemented with 10% fetal bovine serum (PAN-Biotech, catalog no. P40-39500) and 1% penicillin/streptomycin (Gibco, catalog no. 15140122). Rat primary aortic endothelial cells (ECs) were obtained from the Cell Biologics (catalog no. M1266) and cultured in complete rat endothelial cell medium (Cell Biologics, catalog no. M1266-Kit) supplemented with 0.1% vascular endothelial growth factor, 1% l-glutamine, 1% antibiotic-antimycotic solution, and 2% fetal bovine serum. ECs were passaged every 3 to 4 days when confluency reached >80%. Cells were treated with cGAMP (Cambridge Bioscience Ltd, catalog no. G016499-1 MG), cGMP (Merck, G6129), cAMP (Merck, A6885), or lipopolysaccharide (LPS; Merck, L2630). Alternatively, cells were transfected with G3-YSD (Invitrogen, catalog no. Tlrl-ydna), pTRIP-CMV-Puro-2A-cGAS (Addgene plasmid no. 102612), pTRIPZ shcGAS-Has (Addgene plasmid no. 128175), pTRIPZ shNS (Addgene plasmid no. 127696), MRP1 small interfering RNA (siRNA) (Horizon Discovery, J-091531-09-0002), LRRC8A siRNA (Horizon Discovery, J-090547-09-0002), STING siRNA (Horizon Discovery, J-104499-07-0002), TBK1 siRNA (Horizon Discovery, J-10406-10-0002), or nontargeting siRNA (Horizon Discovery, D-001810-01-05) using Lipofectamine 3000 Reagent (Invitrogen, catalog no. L3000008)

### Immunoblotting

After incubation cells or tissue were lysed in sample buffer containing 50 mmol/L Tris-HCl pH 6.8, 2% w/v sodium dodecyl sulfate, 10% glycerol, 0.0025% w/v bromophenol blue, and 5% β-mercaptoethanol. Protein samples were then loaded and resolved on Mini-PROTEAN TGX Gels and then transferred onto polyvinylidene fluoride membranes (BioRad) using a Trans-Blot Turbo Transfer System (BioRad). The polyvinylidene fluoride membranes were then blocked with 10% nonfat dry milk or BSA in PBS with 0.1% v/v Tween (PBS-T) for 1 hour, and then incubated with the primary antibodies. Membranes were developed with the Pierce ECL detection system (Thermo-Fisher Scientific) and signal detected using photographic film (GE Healthcare, catalog no. 28-9068-37) and an automatic processor (Fuji RG II). Samples were immunoblotted for Phospho-VASP (Ser239; Cell Signaling, No. 3114), Phospho-VASP (Ser157; Cell Signaling, no. 84519), cGAS (Invitrogen, no. PA5-76367), Phospho-TBK1 (Ser172; Cell Signaling, no. 5483), TBK1 (Cell Signaling, no. 3013), Phospho-STING (Ser365; Cell Signaling, no. 72971), CD31 (Abcam, no. ab28364), MRP1 (Santa Cruz, no. sc-18835), LRRC8A (Santa Cruz, no. sc-517113), GAPDH (Cell Signaling, no. 2118), and β-actin (Sigma-Aldrich, no. A5316).

### PKGI Activity Assay

The activation of PKG by cGAMP or cGMP was assessed using the PRKG1 (PKGI) Kinase Enzyme System (Promega, catalog no. V4248) and ADP-Glo Kinase Assay (Promega, catalog no. V9101). In brief, the PRKG1 was prepared according to the instructions. A gradient of cGAMP or cGMP concentrations was added to corresponding wells in a 96-well plate. PRKG1 reaction buffer was then added into each well mixed with ATP/substrate and incubated for 60 minutes at room temperature. After incubation, ATP was depleted by adding ADP‐Glo Reagent to each well with incubation for 40 minutes at room temperature. Finally, to detect ADP in each well, Kinase Detection Reagent was added and incubated for 60 minutes at room temperature. The luminescence of each well was recorded using a GloMax Discover microplate reader (Promega, catalog no. GM3000). The percentage of PKGI activity was determined by comparison with kinase that had been fully activated.

### Measuring cGAMP

Release of cGAMP from cultured ECs was assessed using a 2′,3′-cGAMP ELISA assay (Cayman, no. 501700). The concentration of 2′,3′-cGAMP in cell medium was measured according to the manufacturer’s instructions. In brief, cells were cultured in phenol red-free endothelial medium (Cellbiologics, catalog no. M1266PF). After treatment, medium was extracted and concentrated using a speedvac (MiVac DNA Concentrator, no. DNA10120396). The wells of a 96-well plate precoated with mouse anti-rabbit IgG were rinsed 5 times with wash buffer. Samples were added into wells mixed with 2′,3′-cGAMP-HRP Tracer and 2′,3′-cGAMP antibody and incubated overnight at 4°C. After incubation, wells were washed 5 times to remove unbound reagents and 3,3′,5,5′-tetramethylbenzidine substrate solution was added to each well, followed by the HRP stop solution. The absorbance at 450 nm of each well was recorded using a GloMax Discover microplate reader (Promega, catalog no. GM3000).

### Animals

All procedures were performed in accordance with the Home Office Guidance on the Operation of the Animals (Scientific Procedures) Act 1986 in the United Kingdom. Experiments were approved by the King’s College London Animal Welfare and Ethical Review Body. Mice used in this study were on the C57BL/6J genetic background and cGAS^–/–^ mice were purchased from the Jackson Laboratory (Strain no. 026554). Male wild-type or littermate cGAS^–/–^ mice were used in studies at 12 to 15 weeks of age. No animals were excluded from this study.

### Isolation of Mouse Aorta

Dissected mouse thoracic aorta was transferred into a 15-cm cell culture dish. After surrounding tissues and fat were carefully removed, aorta was cut evenly into 4 pieces and then each transferred into a separate well within a 12-well plate containing 1 mL prewarmed Krebs buffer (126 mmol/L NaCl, 2.5 mmol/L KCl, 25 mmol/L NaHCO_3_, 1.2 mmol/L NaH_2_PO_4_, 1.2 mmol/L MgCl_2_, 2.5 mmol/L CaCl_2_, pH 7.2). Aortic segments were transfected with cGAMP, G3-YSD, or LPS for the desired time. After transfection, tissue pieces were then added to 50 µL of sample buffer. For denuded aorta preparations, the lumen of vessels was gently rubbed with fine steel wire to remove the endothelium, while leaving the smooth muscle intact.

### Myography

Dissected mouse thoracic aorta was isolated and cut into rings of ≈3-mm width in ice-cold Krebs buffer (126 mmol/L NaCl, 2.5 mmol/L KCl, 25 mmol/L NaHCO_3_, 1.2 mmol/L NaH_2_PO_4_, 1.2 mmol/L MgCl_2_, 2.5 mmol/L CaCl_2_, pH 7.2). The whole procedure was done carefully to avoid stretch-induced injury to the endothelium. Aortic rings were then mounted into a Multi Wire Myograph System (DMT 620M) containing Krebs buffer. To maintain the aortic rings, Krebs buffer was kept at 37°C, with mixed gas (95% O_2_, 5% CO_2_) continuously bubbling through the chamber. Vessels were stretched to the optimal pretension using the normalization module. After 60 minutes of equilibration, the arteries were “woken up” with 60 mmol/L KCl–induced contraction followed by 3 washes. After 15 minutes resting, aortic rings were transfected with 500 ng/mL G3-YSD or cGAMP for 3 hours. In addition, in some experiments, vessels were pretreated with 5 μmol/L 1*H*-[1,2,4]oxadiazolo[4,3-a]quinoxalin-1-one or 20 μmol/L 4-(2-butyl-6,7-dichloro-2-cyclopentyl-indan-1-on-5-yl) oxobutyric acid for 3 hours before transfection, or 50 μmol/L Rp-8-Br-cGMPS was added 1 hour before the end of the transfection. After transfection, a cumulative half-log dose-dependent response to phenylephrine from 10^–10^ to 10^–4^ mol/L was applied to mock, G3-YSD, or cGAMP transfected aorta. Vasoconstriction measurements were made by determining the continuous responses of phenylephrine-constricted vessels in the presence or absence of cGAMP or G3-YSD. In addition, to verify the removal of endothelium from denuded vessels, the response to acetylcholine (10^–6^ mol/L, Sigma-Aldrich) was examined. The endothelium was considered removed when the degree of vascular relaxation to acetylcholine was <5% in constricted vessels. For mesenteric vessels these were constricted with a cumulative dose of U46619 (10^–11^–10^–5^ mol/L).

### Telemetric Blood Pressure Monitoring

Data were analyzed in a blinded manner and no mice were excluded. Mice were anesthetized with 2% isoflurane (Centaur Services) and provided 0.5 L of oxygen per minute with pre- and postoperative analgesia (buprenorphine, 0.1 mg per kg of body weight; Abbot Laboratories). A radiotelemetry probe catheter (TA11PA-C10, Data Science International) was implanted in the aortic arch through the left carotid artery. After at least 7 days of recovery, mice housed individually in cages were placed above telemetric receivers. Blood pressure was recorded by scheduled sampling for 10 seconds every 5 minutes (Dataquest LabPRO Acquisition system version 3.01; Data Sciences International). After the baseline blood pressure was monitored, mice were given G3-YSD (1.6 mg/kg), G3-YSD-control (1.6 mg/kg), cGAMP (6 mg/kg), or LPS (9 mg/kg). For transfection, G3-YSD, G3-YSD-control, or cGAMP was mixed with in vivo JetPEI (Polyplus) reagent according to the manufacturer’s instructions and then administered through intraperitoneal injection.

### Statistical Analysis

Data are shown as mean±SEM. Differences between groups were assessed using ANOVA followed by the Tukey post hoc test. All datasets had a normal distribution, as confirmed using the Shapiro-Wilk normality test. Results were considered significant at a 5% significance level.

## RESULTS

### cGAS/cGAMP Can Activate VSMC PKGI

**Figure 1.**
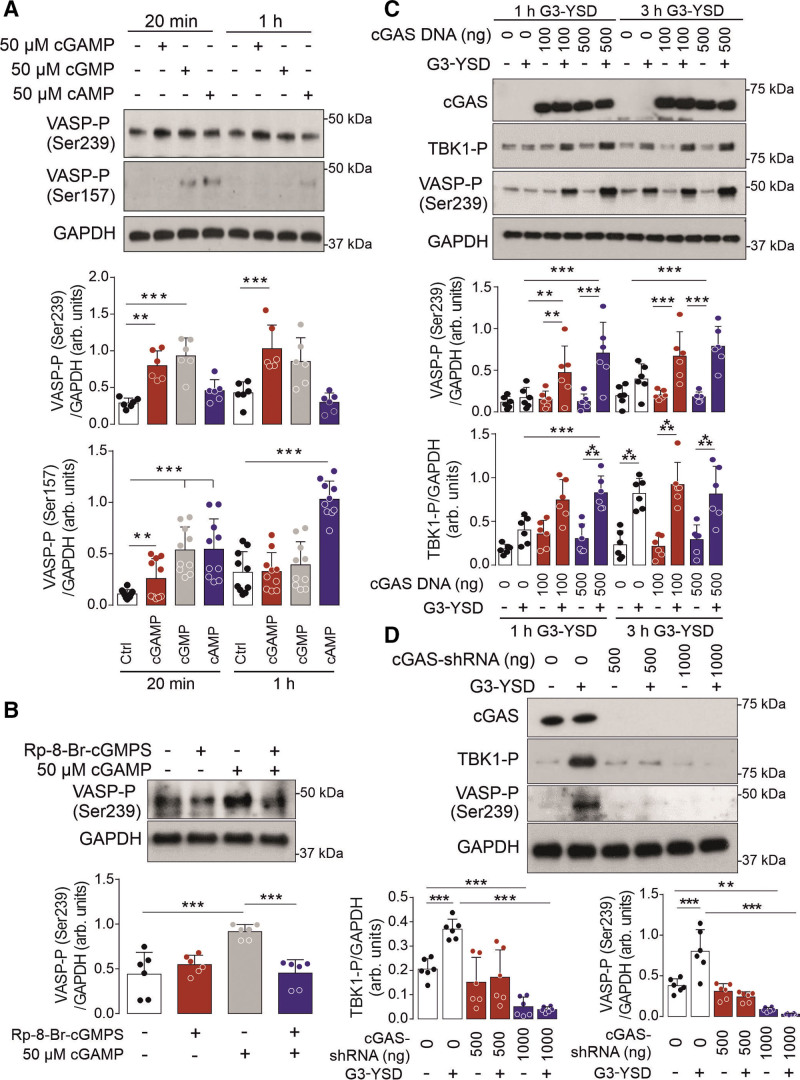
**cGAS/cGAMP can activate vascular smooth muscle cell PKGI. A**, VASP Ser239 and Ser157 phosphorylation (n=6, 10) in vascular smooth muscle cells treated with cGMP, cAMP, or cGAMP. **B**, VASP Ser239 phosphorylation in vascular smooth muscle cells in response to cGAMP treatment after PKGI inhibition with Rp-8-Br-cGMPS (n=6). **C**, TBK1 and VASP phosphorylation in vascular smooth muscle cells with or without cGAS overexpression and in the presence or absence of G3-YSD (n=6). **D**, TBK1 and VASP Ser239 phosphorylation in vascular smooth muscle cells with or without knockdown of cGAS overexpression and in the presence or absence of G3-YSD (n=6). **P<0.01; ***P<0.005. Comparisons were made using 1-way ANOVA (**A**) or 2-way ANOVA (**B**, **C**, **D**) followed by the Tukey post hoc test. arb. indicates arbitrary; cGAMP, cyclic GMP-AMP; cGAS, cyclic GMP-AMP synthase; Ctrl, control; PKGI, cGMP-dependent protein kinase 1; shRNA, short hairpin RNA; TBK1, TANK-binding kinase 1; and VASP, vasodilator stimulated phosphoprotein.

On the basis of the composition of 2′,3′-cGAMP, we hypothesized that this dicyclic nucleotide may activate PKA (cAMP-dependent protein kinase) or PKGI. This is consistent with these kinases being able to accommodate noncanonical cyclic nucleotides, including larger analogues.^15,16^ To test this hypothesis, we assessed the site-specific phosphorylation of their substrate vasodilator-stimulated phosphoprotein (VASP) in cultured VSMCs.^17^ Here we found that cGAMP treatment induced phosphorylation of the PKGI-specific site Ser239, but not the PKA site Ser157 (Figure 1A). The phosphorylation of VASP at Ser239 by cGAMP-dependent PKGI activation was substantiated, because this was attenuated in cells pretreated with the PKGI-specific inhibitor Rp-8-Br-cGMPS (Figure 1B). To assess whether endogenous formation of cGAMP could also activate PKGI, VSMCs were transfected with the cGAS activator G3-YSD.^18^ After 3 hours transfection, VASP Ser239 phosphorylation was enhanced and further potentiated in cells where cGAS was overexpressed (Figure 1C). The phosphorylation of VASP also mirrored TBK1 (TANK-binding kinase 1) phosphorylation, consistent with this process being cGAMP-dependent.^19,20^ This was further substantiated because VASP Ser239 phosphorylation mediated by G3-YSD was attenuated in cells deficient in cGAS (Figure 1D).

### cGAMP Directly Activates PKGI

**Figure 2.**
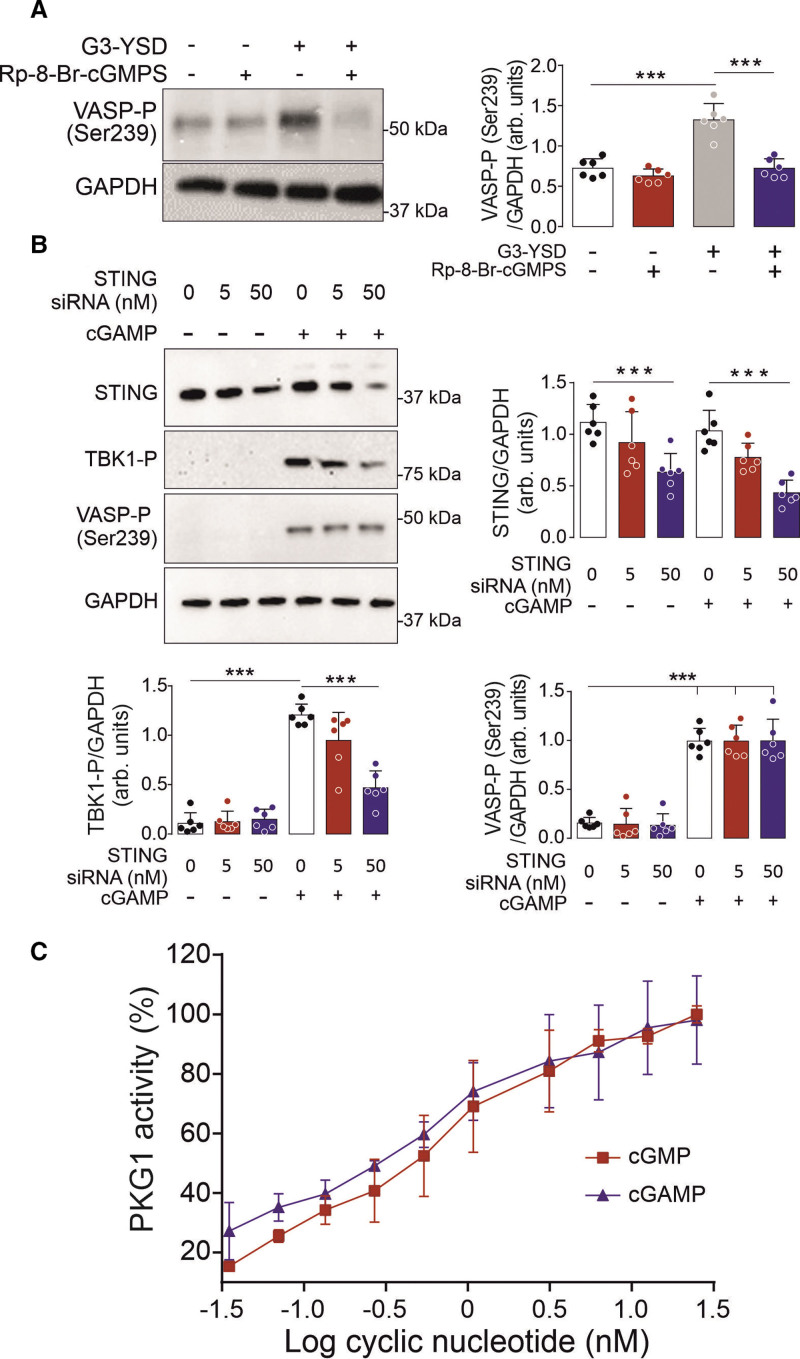
**Direct PKGI activation by cGAMP. A**, VASP Ser239 phosphorylation in vascular smooth muscle cells in response to G3-YSD treatment after PKGI inhibition with Rp-8-Br-cGMPS (n=6). **B**, TBK1 and VASP Ser239 phosphorylation in cells with or without knockdown of STING in the presence or absence of cGAMP (n=6). **C**, The activity of recombinant PKGI treated with cGMP or cGAMP (n=4). ***P<0.005. Comparisons were made using 1-way ANOVA (**C**) or 2-way ANOVA (**A**, **B**) followed by the Tukey post hoc test. arb. indicates arbitrary; cGAMP, cyclic GMP-AMP; PKGI, cGMP-dependent protein kinase 1; siRNA, small interfering RNA; STING, stimulator of interferon genes; TBK1, TANK-binding kinase 1; and VASP, vasodilator stimulated phosphoprotein.

To confirm that cGAS was able to activate PKGI in VSMCs, the kinase was targeted using the specific inhibitor Rp-8-Br-cGMPS. Here, Rp-8-Br-cGMPS was found to attenuate VASP Ser239 phosphorylation induced by G3-YSD, thus substantiating PKGI activation by cGAS (Figure 2A). Because STING is the main mediator of cGAMP signaling, we next assessed its potential role in the activation of PKGI. Because STING can regulate the formation of reactive oxygen species,^21^ we assessed PKGI-α cysteine 42–dependent disulfide dimerization that is known to mediate direct cGMP-independent kinase activation.^22,23^ Here cGAMP was found not to influence cysteine 42–dependent disulfide dimerization, thus ruling out this mechanism of PKGI activation (Figure S1A). Furthermore, loss in STING or TBK1 did not affect VASP Ser239 phosphorylation in cells transfected with G3-YSD (Figure 2B; Figure S1B). This indicates that cGAMP is likely to directly activate PKGI, which was further investigated using an in vitro kinase assay. Here cGAMP was found to directly activate PKGI that mirrored activation by cGMP (Figure 2C). Together these experiments identify a novel mechanism of direct PKGI activation by cGAMP that is independent of STING.

### cGAS Activation Mediates Vasorelaxation

**Figure 3.**
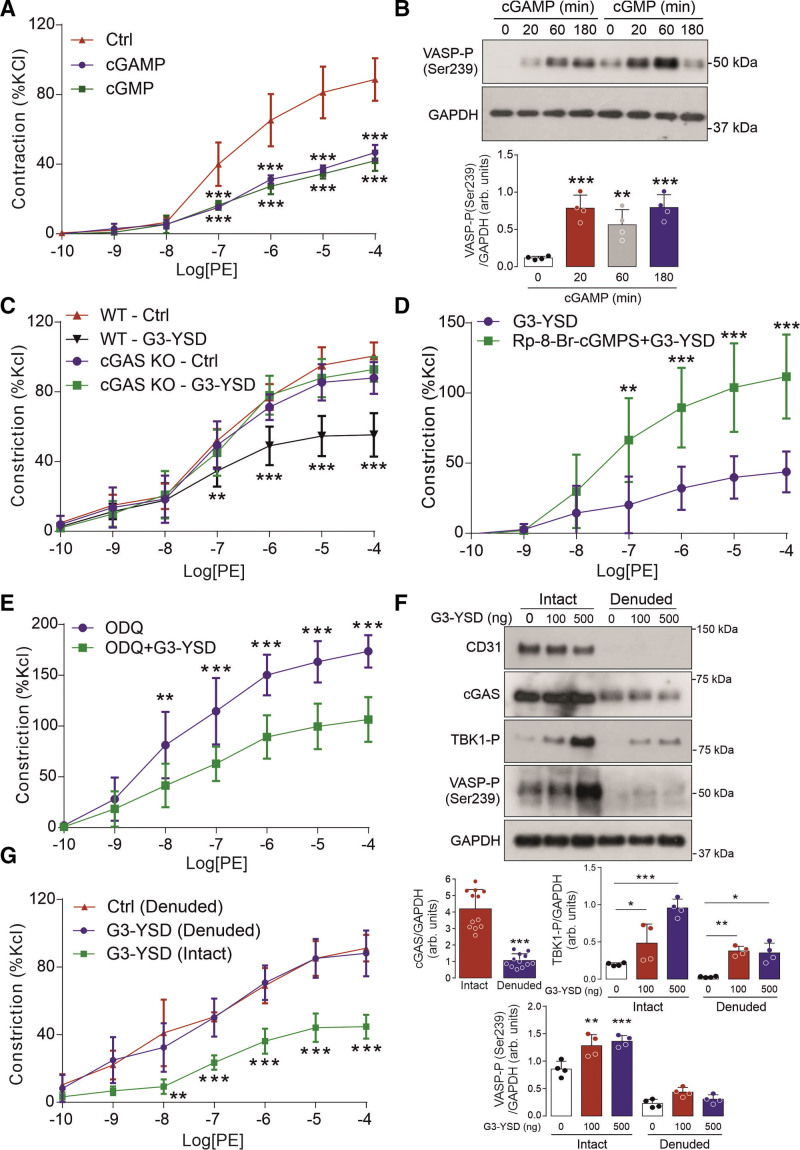
**cGAS activation within the endothelium induces cGAMP-dependent vessel relaxation. A**, Vasoconstriction to phenylephrine of isolated aorta treated with cGMP or cGAMP (n=4). **B**, VASP Ser239 phosphorylation in mouse aorta treated with cGAMP (n=4). **C**, Vasoconstriction to phenylephrine of isolated aorta treated with G3-YSD from WT or cGAS littermate mice (n=6). **D**, Vasoconstriction to phenylephrine of isolated aorta treated with G3-YSD in the presence or absence of the PKGI inhibitor RP-8-Br-cGMPS (n=5–6). **E**, Vasoconstriction to phenylephrine of isolated aorta treated with G3-YSD in the presence or absence of the soluble guanylate cyclase inhibitor ODQ (n=8). **F**, The abundance of cGAS (n=12), and VASP and TBK1 phosphorylation, as well, in intact and denuded mouse aorta treated with G3-YSD (n=4). **G**, Vasoconstriction to phenylephrine of intact or denuded aorta treated with G3-YSD (n=4–6). *P<0.05; **P<0.01; ***P<0.005. Comparisons were made using 1-way ANOVA (**B**, **F**) or 2-way ANOVA (**A**, **C**, **D**, **E**, **G**) followed by the Tukey post hoc test. cGAMP indicates cyclic GMP-AMP; cGAS, cyclic GMP-AMP synthase; Ctrl, control; KO, knockout; ODQ, 1*H*-[1,2,4]oxadiazolo[4,3-a]quinoxalin-1-one; PE, phenylephrine; TBK1, TANK-binding kinase 1; VASP, vasodilator stimulated phosphoprotein; and WT, wild type.

Consistent with a direct mode of PKGI activation, transfection with cGAMP attenuated the vasoconstriction of isolated thoracic aortas and mesenteric arteries (Figure 3A; Figure S1C), and enhanced VASP Ser239 phosphorylation, as well (Figure 3B). This mechanism was further substantiated because G3-YSD failed to attenuate the constriction of aorta and mesenteric arteries taken from male cGAS^–/–^ mice (Figure 3C; Figure S1D and S1E).^24^ This effect of G3-YSD on the vasoconstriction of mesenteries was also comparable in vessels isolated from female mice (Figure S1F). In addition, this process was found to be dependent on PKGI activity (Figure 3D), but independent of cGMP/soluble guanylate cyclase (Figure 3E), thus consistent with the direct cGAMP-dependent activation of PKGI. However, activation of PKGI and the attenuation in vasoconstriction observed on stimulation of cGAS by G3-YSD was found to be endothelium-dependent because this process was absent in denuded vessels (Figure 3F and 3G). This was also consistent with the expression of cGAS that was also largely found within the endothelium. In summary, these findings show that activation of cGAS mediates vessel relaxation that is dependent on the intact endothelium and PKGI activity, but independent of cGMP/soluble guanylate cyclase.

### cGAMP Transport Mediates PKGI Activation

**Figure 4.**
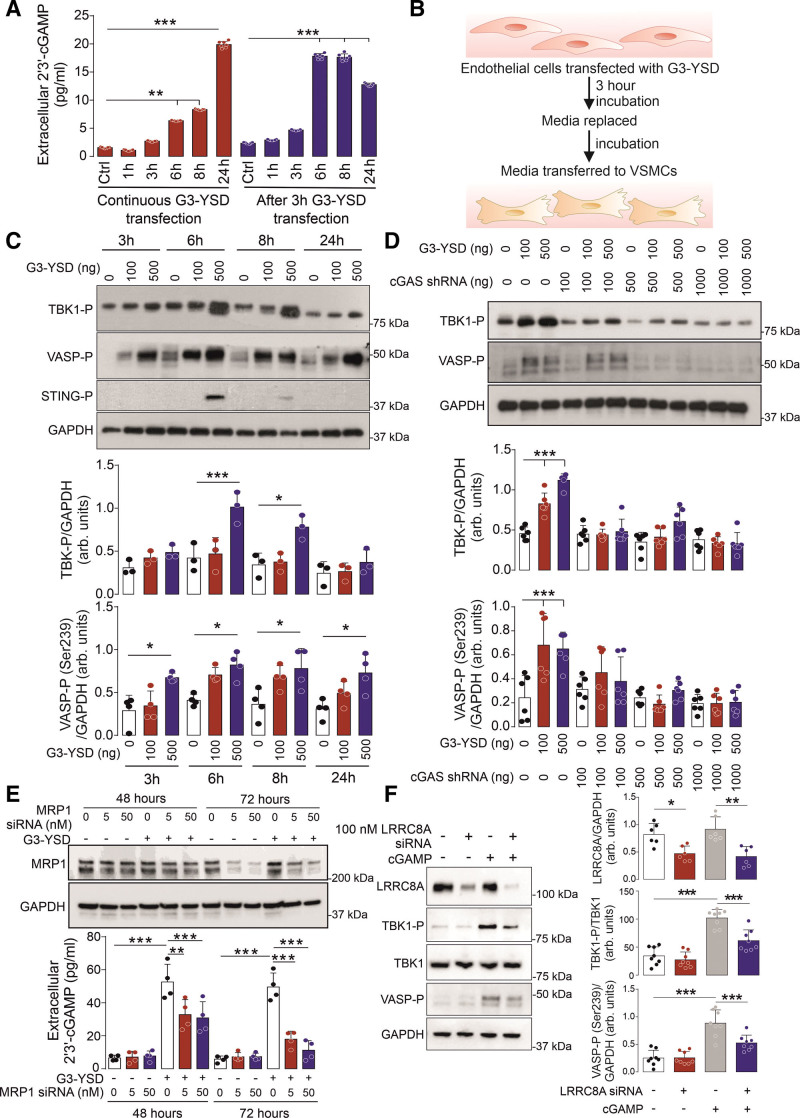
**Transport of cGAMP from the endothelium to vascular smooth muscle cells underlies cGAS-dependent PKGI activation. A**, Detection of cGAMP in the media of ECs transfected with G3-YSD (n=7). **B**, Strategy used to test whether cGAMP released from vascular endothelial cells can activate PKGI in rat aortic smooth muscle cells. **C**, TBK1 and VASP Ser239 phosphorylation in VSMCs exposed to media taken from ECs treated with or without G3-YSD (n=3–4). **D**, TBK1 and VASP Ser239 phosphorylation in VSMCs exposed to media taken from ECs with or without knockdown of cGAS (n=6). **E**, Detection of cGAMP in the media of ECs transfected with G3-YSD and with or without knockdown of MRP1 (n=4). **F**, TBK1 and VASP Ser239 phosphorylation in VSMCs treated with cGAMP and with or without the knockdown of the volume-regulated anion channel subunit LRRC8A (n=6, 8). *P<0.05; **P<0.01; ***P<0.005. Comparisons were made using 1-way ANOVA (**A**, **C**) or 2-way ANOVA (**D**, **E**, **F**) followed by the Tukey post hoc test. arb. indicates arbitrary; cGAMP, cyclic GMP-AMP; cGAS, cyclic GMP-AMP synthase; Ctrl, control; MRP1, multidrug resistance protein 1; PKGI, cGMP-dependent protein kinase 1; shRNA, short hairpin RNA; siRNA, small interfering RNA; STING, stimulator of interferon genes; TBK1, TANK-binding kinase 1; VASP, vasodilator stimulated phosphoprotein; and VSMC, vascular smooth muscle cell.

To further substantiate the role of the endothelium in mediating cGAMP-dependent vessel relaxation we assessed the release of this cyclic nucleotide from aortic ECs. Here a time-dependent increase in extracellular cGAMP was detected after activation of cGAS following transfection of ECs with G3-YSD (Figure 4A). To establish whether the cGAMP released from ECs could activate PKGI in VSMCs, media from ECs stimulated with G3-YSD were added to VSMCs (Figure 4B). Here media from G3-YSD-transfected ECs led to enhanced TBK1 and VASP phosphorylation in VSMCs (Figure 4C). This is consistent with the release of cGAMP from ECs and its uptake into VSMCs where it activates PKGI and TBK1. This was further substantiated because media from ECs deficient in cGAS failed to induce phosphorylation of TBK1 and VASP in VSMCs (Figure 4D; Figure S2A). To establish the mechanism of cGAMP export, we targeted the transporter that is already associated with this process, namely MRP1.^25^ Here the inhibition or knockdown of MRP1 led to loss in the release of cGAMP in ECs treated with G3-YSD, thus substantiating its role in mediating the export of this dicyclic nucleotide (Figure 4E; Figure S2B and S2C). To identify the mechanism of cGAMP import into VSMCs, we targeted channels associated with its transport, namely SLC19A1, P2X7R, and VRAC.^26–28^ Here only inhibition of VRAC was effective in preventing cGAMP-dependent TBK1 phosphorylation (Figure S2D–S2F). The importance of VRAC in mediating cGAMP import was further substantiated, because the knockdown of the key subunit LRRC8A also attenuated TBK1 phosphorylation, and VASP Ser239 phosphorylation in cells treated with this cyclic dinucleotide, as well (Figure 4F). Furthermore, inhibition of VRAC attenuated the ability of cGAMP to limit vasoconstriction to phenylephrine in isolated thoracic aorta (Figure S2G and S2H). Together these experiments show that cGAMP generated by cGAS within the endothelium is exported by MRP1 and taken up into VSMCs by VRAC where it can then activate PKGI.

### cGAS Mediates Lowering of Blood Pressure

**Figure 5.**
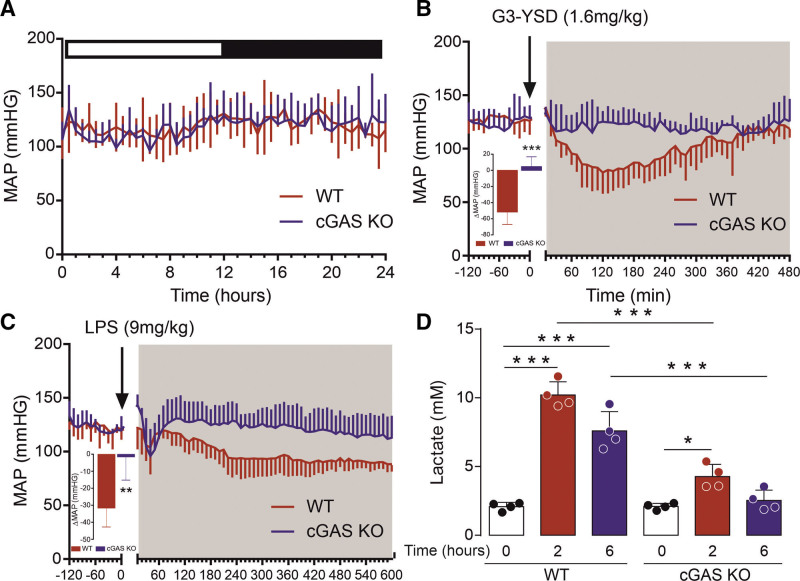
**Activation of cGAS mediates lowering of blood pressure and tissue hypoperfusion during sepsis. A**, Resting blood pressure in WT and cGAS^–/–^ littermate mice (n=4). **B**, Blood pressure in WT and cGAS^–/–^ littermate mice after administration of G3-YSD (n=5). **C**, Blood pressure in WT and cGAS^–/–^ littermate mice after administration of LPS (n=6). **D**, Plasma lactate in WT and cGAS^–/–^ littermate mice after administration of LPS (n=4). *P<0.05; **P<0.01; ***P<0.005. Comparisons were made using 1-way ANOVA (**A**, **B**, **C**) or 2-way ANOVA (**D**) followed by the Tukey post hoc test. cGAS indicates cyclic GMP-AMP synthase; KO, knockout; LPS, lipopolysaccharide; MAP, mean arterial pressure; and WT, wild type.

Because activation of PKGI by cGAMP led to vasodilation, the effect of this process on blood pressure was then investigated. To activate cGAS within the vasculature, G3-YSD was administered to mice with use of an in vivo transfection reagent. Consistent with cGAS activation, an increase in TBK1 and VASP phosphorylation was observed in thoracic aorta isolated from wild-type (WT) mice, but not cGAS^–/–^ mice given G3-YSD (Figure S3A). To test the effect of cGAS activation on blood pressure, we used mice implanted with telemetry probes. In WT mice, a pronounced and sustained decrease in blood pressure (–51.6±6.9 mm Hg) was observed after transfection with G3-YSD, which did not occur in cGAS^–/–^ mice (4.7±5.4 mm Hg) (Figure 5A and 5B, Figure S3B–S3I). This was further substantiated because transfection with G3-YSD-control (3 bases that differ from G3-YSD, preventing it from activating cGAS) had no effect on blood pressure (Figure S3J–S3N), whereas transfection with cGAMP led to rapid and pronounced hypotension (Figure S3O–S3S). This highlights a novel function of cGAS in regulating blood pressure, which is consistent with cGAMP-dependent PKGI activation. In addition, this process is likely to be important during infection due to the activation of cGAS.^29^ Here additional studies were conducted using a model of lipopolysaccharide (LPS)-induced inflammation. The endotoxin bacterial LPS is well-known to induce systemic inflammation and is the most potent microbial mediator implicated in the pathogenesis of sepsis.^30,31^ In ECs exposed to LPS, we observed an increase in the release of cGAMP (Figure S4A). This observation concurs with a study showing that LPS activates endothelial-localized cGAS due to release of mitochondrial DNA.^32^ Consistent with enhanced formation of endothelial-derived cGAMP, telemetered WT mice that were given LPS had a substantial drop in blood pressure (–31.5±4.6 mm Hg), whereas only a marginal effect was observed in cGAS^–/–^ mice (–1.5±5.6 mm Hg; Figure 5C; Figure S4B–S4E). In addition, enhanced phosphorylation of VASP and TBK1 observed in the aorta after administration of LPS was blunted in cGAS^–/–^ mice (Figure S4F). These observations also correlated with levels of plasma lactate, a marker of disease severity and tissue hypoperfusion (Figure 4D). Together these results show that activation of cGAS mediates the reduction of blood pressure and that this process contributes to hypotension and tissue hypoperfusion during sepsis.

## DISCUSSION

Here we have identified PKGI as a novel direct sensor for cGAMP, which provides a new mechanism of crosstalk between inflammation and blood pressure regulation. Although the direct activation of STING has been well characterized,^4,5^ the structural similarity of cGAMP to cAMP and cGMP led us to hypothesize that this dicyclic nucleotide may also bind and directly activate PKA or PKGI.^5,33^ By analyzing specific phosphorylation sites on VASP in VSMCs we were able to conclude that cGAMP did activate PKGI, but not PKA. The activation of PKGI by cGAMP was found to be independent of the canonical STING pathway and instead due to direct kinase activation. Although cGAMP is a large cyclic nucleotide, these findings are consistent with the direct activation of PKGI by noncanonical cyclic nucleotides that include dimeric forms of cGMP.^15,16^

This mode of PKGI activation by cGAS limited vessel constriction to phenylephrine that was independent of the nitric oxide/cGMP pathway, thus consistent with direct cGAMP-dependent kinase activation. However, the activation of PKGI by cGAS was found to be largely dependent on the endothelium. This mirrors the canonical pathway of kinase activation that is also dependent on the endothelium, because this provides the source of the gaseous transmitter nitric oxide needed to stimulate formation of cGMP within the vascular smooth muscle.^14^ The important role for endothelial-derived cGAMP is also consistent with higher cGAS expression within the endothelium compared with the vascular smooth muscle. This process of EC-VSMC crosstalk provides a mechanism to sense intravascular circulating pathogens and transduce this into a change in vessel tone. This includes sensing circulating bacterial and viral pathogens that will interact with the endothelium and initiate the innate immune response, which when systemic can lead to the development of sepsis.^34^ This is certainly consistent with evidence that the bacterial endotoxin LPS induces mitochondrial DNA release into the cytosol of ECs that activates cGAS.^32^ This process of cGAS activation was found to suppress EC proliferation through the downregulation of YAP1 signaling. In addition, in retinal microvascular ECs, LPS induces release of mitochondrial DNA that drives the activation of noninfectious inflammation through activation of cGAS-STING.^35^ Furthermore, it has been found that, with SARS-CoV-2 viral infection, this can lead to activation of endothelial cGAS-STING signaling due to the release of mitochondrial DNA, which can cause cell death and type I interferon production,^36^ which is likely to contribute to pulmonary EC endotheliitis in critically ill patients.^37^ Also, although cGAS activation was found to induce vasodilation and lower blood pressure, long-term activation of cGAS during aging may in contrast lead to a loss in vascular reactivity.^38^ This is likely to result from endothelial dysfunction due to chronic cGAS activation leading to sustained inflammation and loss in endothelial nitic oxide synthase.

The export of cGAMP from the endothelium was found to be largely dependent on the activity of MRP1. This is consistent with the recent identification of MRP1 as a direct ATP-dependent cGAMP exporter that can regulate intrinsic activation of STING.^25^ Because export of cGAMP limits STING activation, a deficiency in MRP1 leads to enhanced cGAS-dependent autoimmunity in a mouse model of Aicardi-Goutières syndrome. In addition to MRP1, we also found that VRAC was needed for the import of cGAMP into VSMCs. This is consistent with LRRC8A:C/E being a ubiquitous heteromeric channel for transporting cGAMP.^27^ This transport of cGAMP is needed for cGAS-dependent vessel relaxation, and, therefore, it is likely to underly the regulation of blood pressure by this dicyclic nucleotide. Because these findings are based on studies using pharmacological inhibitors, future studies should further investigate the importance of MRP1 and VRAC in regulating cGAS/cGAMP-dependent blood pressure using conditional knockout mice. Nonetheless this mechanism of cGAMP-dependent PKGI activation is consistent with the marked decrease observed in blood pressure in WT mice given G3-YSD, an effect that was absent in cGAS knockout mice. In addition, because G3-YSD–dependent attenuation in constriction was comparable in mesenteric arteries isolated from male and female mice, it is likely that cGAS activation will have a similar effect on blood pressure in both sexes. The biological importance of this process was further highlighted in a model of sepsis, where cGAS activity was required for the development of hypotension. Therefore, activation of PKGI by cGAMP is an important pathophysiological process because it enables the coupling of blood pressure to cytosolic DNA sensing by cGAS and plays a key role in sepsis, where it mediates hypotension and tissue hypoperfusion. Therefore, our findings describe a new crosstalk between inflammation and blood pressure regulation that is mediated by a novel cGAS/PKGI signaling axis that can be exploited for therapeutic intervention.

## ARTICLE INFORMATION

### Sources of Funding

This study was supported by the British Heart Foundation (RE/18/2/34213, PG/19/65/34574, PG/22/10932, and FS/18/60/34181) and the China Scholarship Council (CSC No.202008320269).

### Disclosures

None.

### Supplemental Material

Figures S1–S4

## Supplementary Material
