# Supplementary material for: Sensing Cytosolic DNA Lowers Blood Pressure by Direct cGAMP-Dependent PKGI Activation
Source: Circulation. 2023 Aug 7;148(13):1023–34. doi: 10.1161/CIRCULATIONAHA.123.065547 (PMC10516174; doi:10.1161/CIRCULATIONAHA.123.065547)
Supplement: Supplementary file 1 [file cir-148-1023-s001.pdf]

SUPPLEMENTAL MATERIAL

Supplemental Figures and Figure Legends

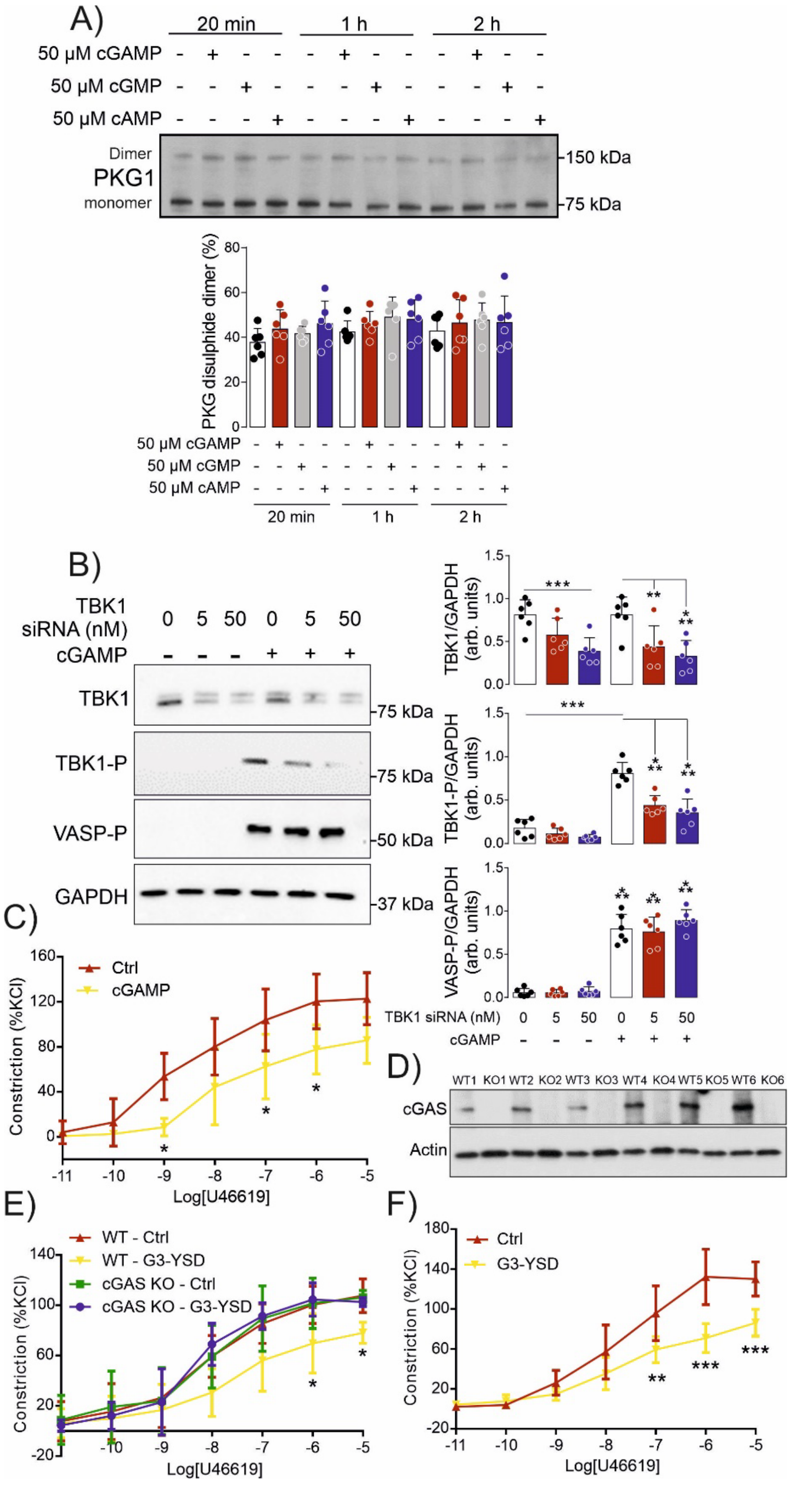

Fig. S1. cGAS/cGAMP mediates the relaxation of mesenteric vessels. a, Treatment with cGAMP does not impact on PKGI disulphide dimerization (n=6). **b,** VASP Ser239 phosphorylation in cells treated with cGAMP is independent of TBK1 (n=6). **c,** The vasoconstriction of isolated mesenteric vessels to U46619 in the presence or absence of cGAMP (n=5). **d,** Validation of cGAS knock out in thoracic aorta from cGAS$^{-/-}$ mice. **e**, The vasoconstriction of isolated mesenteric vessels from WT or cGAS$^{-/-}$ littermate mice to U46619 in the presence or absence of G3-YSD (n=5-6). **f,** The vasoconstriction of isolated mesenteric vessels from female mice to U46619 in the presence or absence of G3-YSD (n=5). *P<0.05; **P<0.01; ***P<0.005. Comparisons were made using 1-way ANOVA (**a**) or 2-way ANOVA (**b, c, e, f**) followed by the Tukey post hoc test.

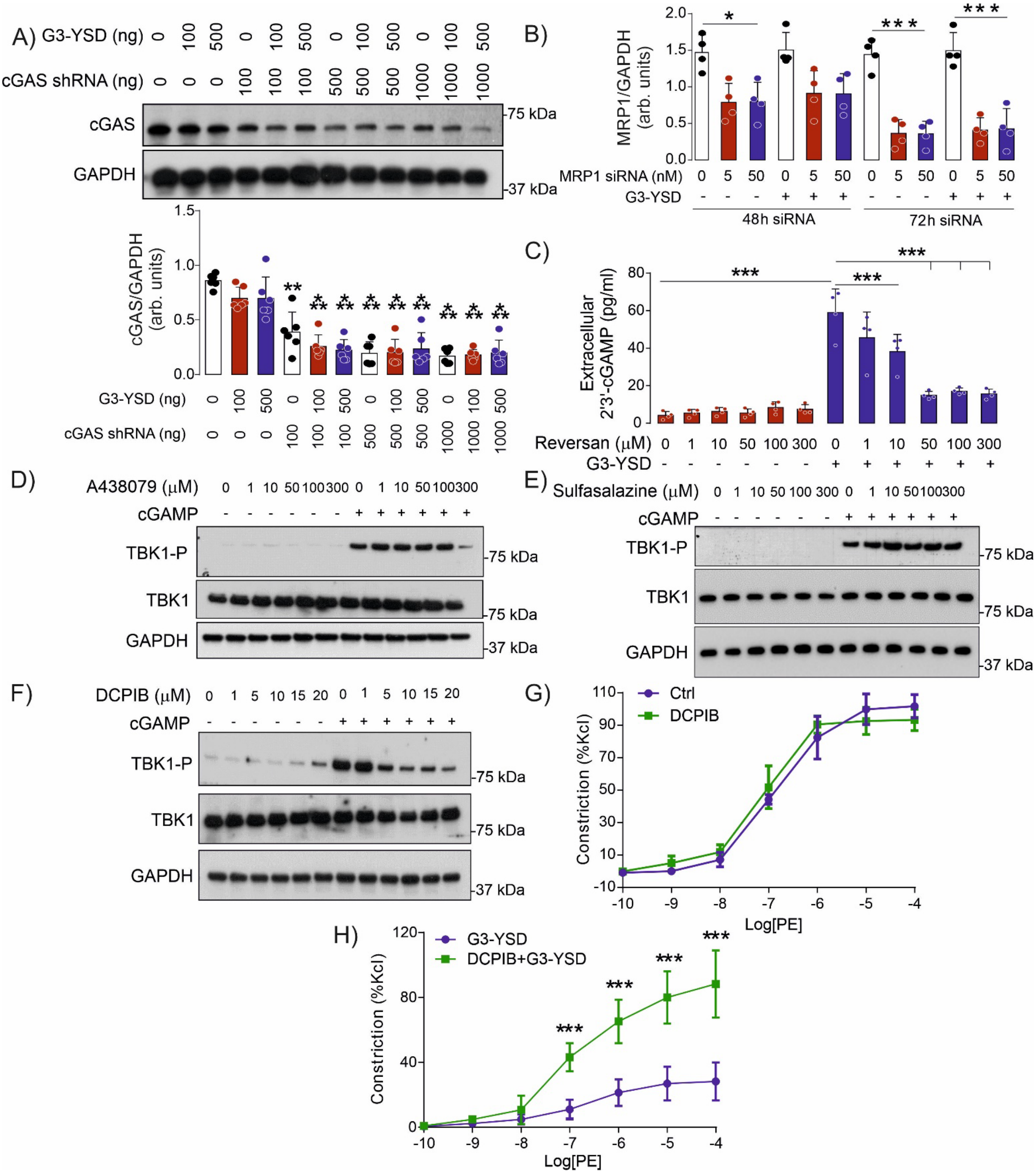

Fig. S2. cGAS mediates vessel relaxation through VRAC-dependent import of cGAMP.
a, Successful knockdown of cGAS expression in endothelial cells (n=6). **b,** Quantification of MRP1 protein abundance (n=4). **c,** The MRP1 inhibitor reversan prevented release of cGAMP from endothelial cells treated with G3-YSD (n=4). Inhibition of P2X7R by A438079, **d,** or SLC19A1 by sulfasalazine, **e,** does not prevent cGAMP-dependent TBK1 phosphorylation. **f**, The VRAC antagonist DCPIB attenuates cGAMP-dependent TBK1 phosphorylation. **g,** The VRAC inhibitor DCPIB does not impact on phenylephrine dependent constriction of isolated aorta (n=3). **h,** The ability of G3-YSD to limit vasoconstriction to phenylephrine is attenuated in the presence of the VRAC inhibitor DCPIB (n=5). *P<0.05; **P<0.01; ***P<0.005. Comparisons were made using 1-way ANOVA (**a, b**) or 2-way ANOVA (**c, g, h**) followed by the Tukey post hoc test.

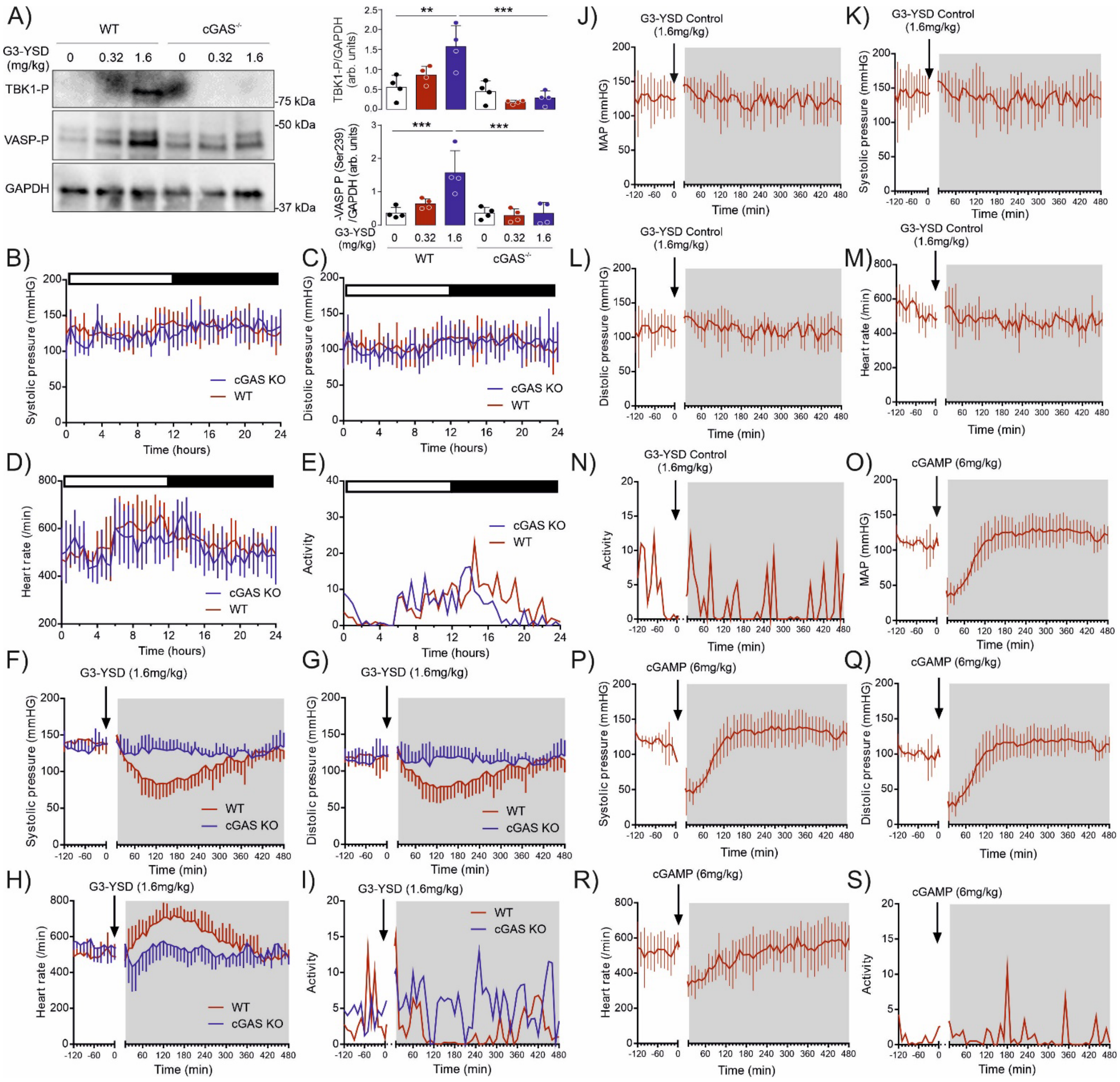

Fig. S3. cGAS activation or administration with cGAMP induces lowering of blood pressure.
a, In vivo administration of G3-YSD led to activation of cGAS and downstream phosphorylation of both TBK1 and VASP in mouse aorta from WT but not cGAS$^{-/-}$ mice (n=4). Baseline telemetry measurements in WT and cGAS$^{-/-}$ mice for **b,** systolic pressure, **c,** diastolic pressure, **d,** heart rate and **e,** activity. Telemetry measurements after administration of G3-YSD for **f,** systolic pressure, **g,** diastolic pressure, **h,** heart rate and **i,** activity (n=5). Telemetry measurements in mice administered G3-YSD-control for **j,** mean arterial pressure **k,** systolic pressure, **l,** diastolic pressure, **m,** heart rate and **n,** activity (n=3). Telemetry measurements in mice administered cGAMP for **o,** mean arterial pressure **p,** systolic pressure, **q,** diastolic pressure, **r,** heart rate and **s,** activity (n=4). **P<0.01; ***P<0.005. Comparisons were made using 2-way ANOVA (**a**) followed by the Tukey post hoc test.

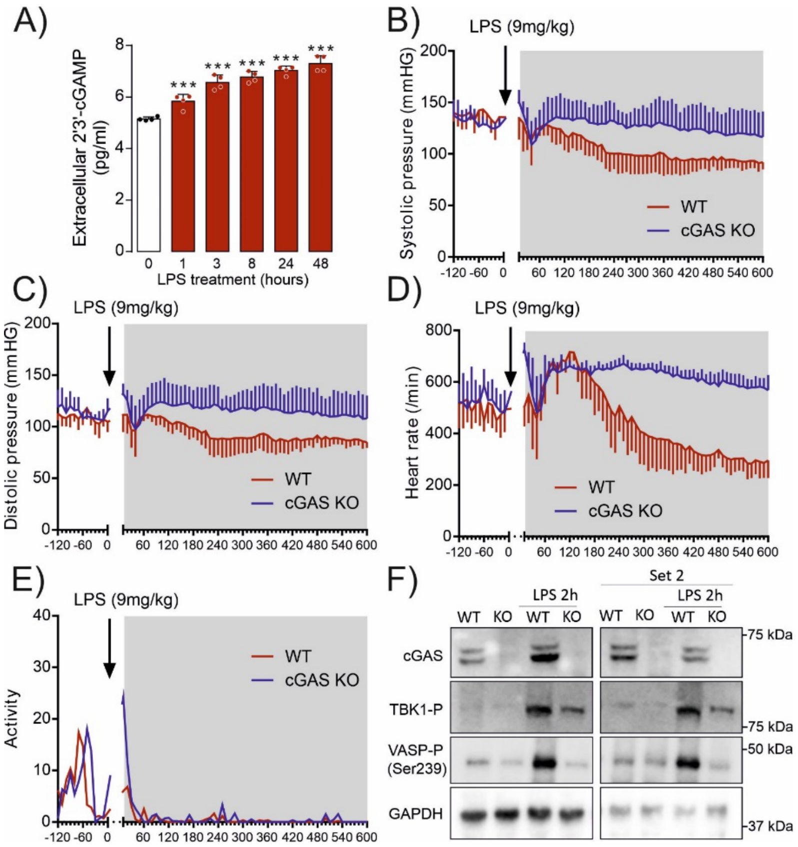

Fig. S4. Treatment with LPS mediates EC release of cGAMP and lowering of blood pressure.
a, Treatment of endothelial cells with LPS leads to a time-dependent accumulation in extracellular cGAMP (n=4). Telemetry measurements in WT and cGAS$^{-/-}$ mice after administration of LPS for **b,** systolic pressure, **c,** diastolic pressure, **d,** heart rate and **e,** activity (n=6). **f,** Enhanced phosphorylation of both VASP and TBK1 in the aorta of mice administered LPS is attenuated in cGAS$^{-/-}$ mice. ***P<0.005. Comparisons were made using 1-way ANOVA (**a**) followed by the Tukey post hoc test.
