# Supplementary material for: Sensing Cytosolic DNA Lowers Blood Pressure by Direct cGAMP-Dependent PKGI Activation
Source: Circulation. 2023 Aug 7;148(13):1023–34. doi: 10.1161/CIRCULATIONAHA.123.065547 (PMC10516174; doi:10.1161/CIRCULATIONAHA.123.065547)

Full unedited blots for Figure 1

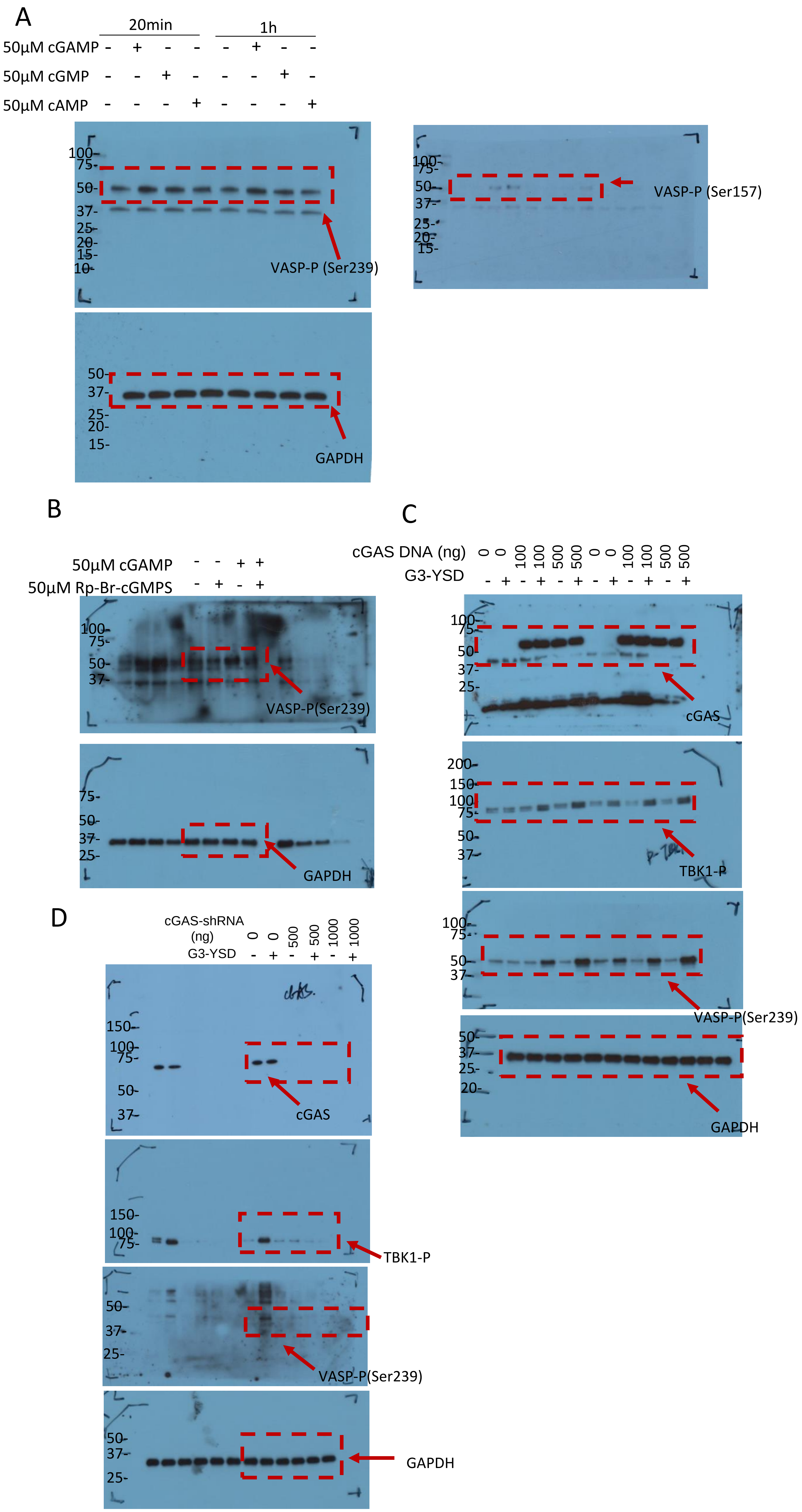

Full unedited blots for Figure 2

A

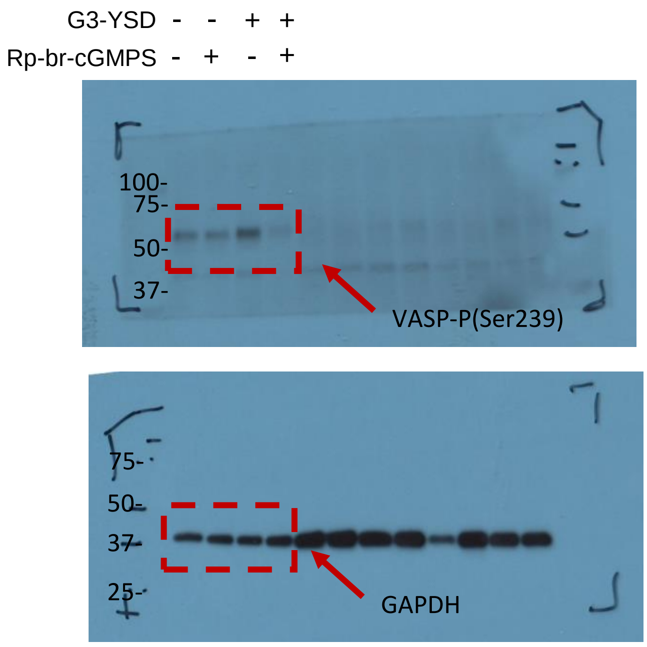

B

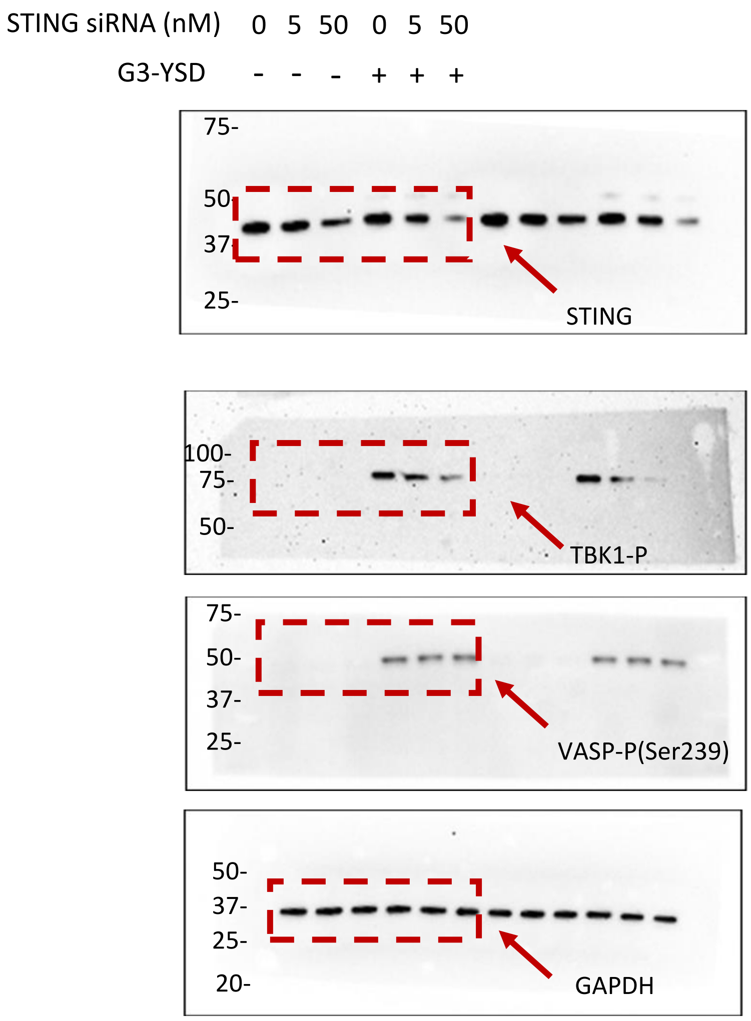

Full unedited blots for Figure 3

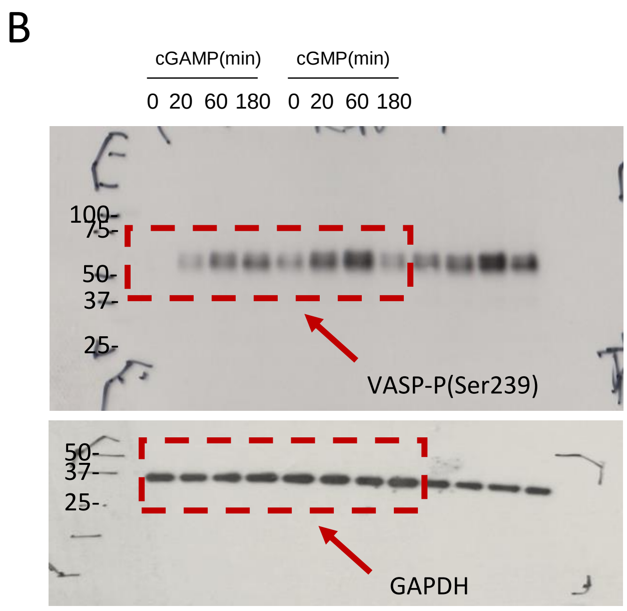

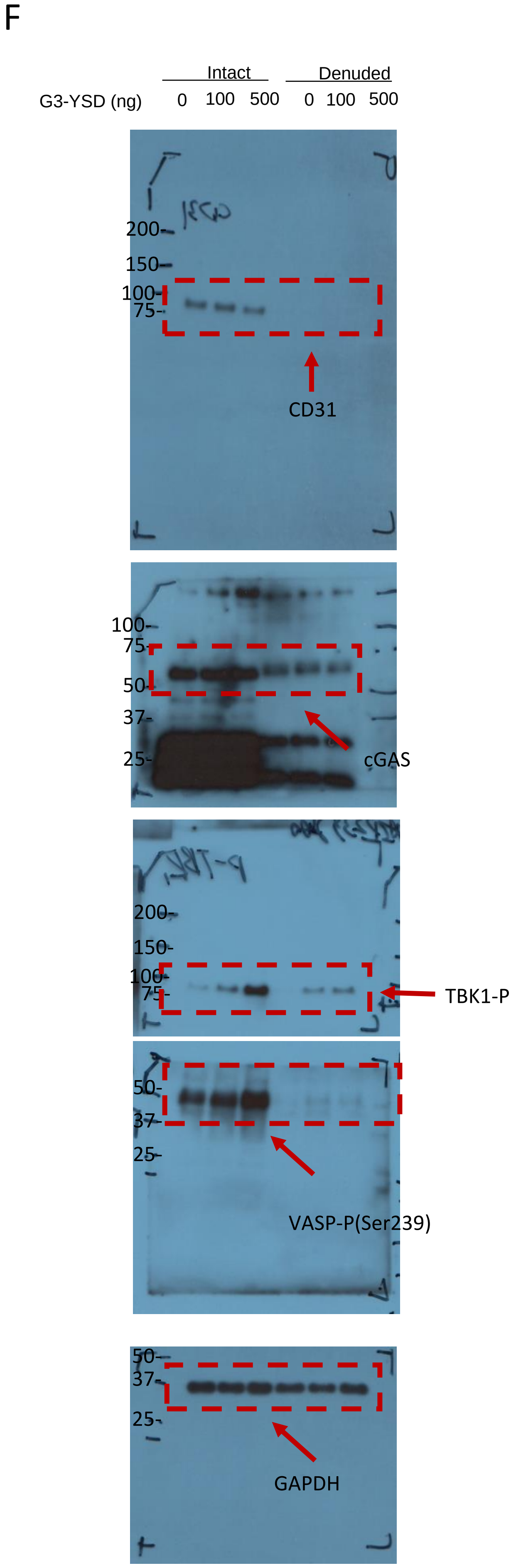

Full unedited blots for Figure 4

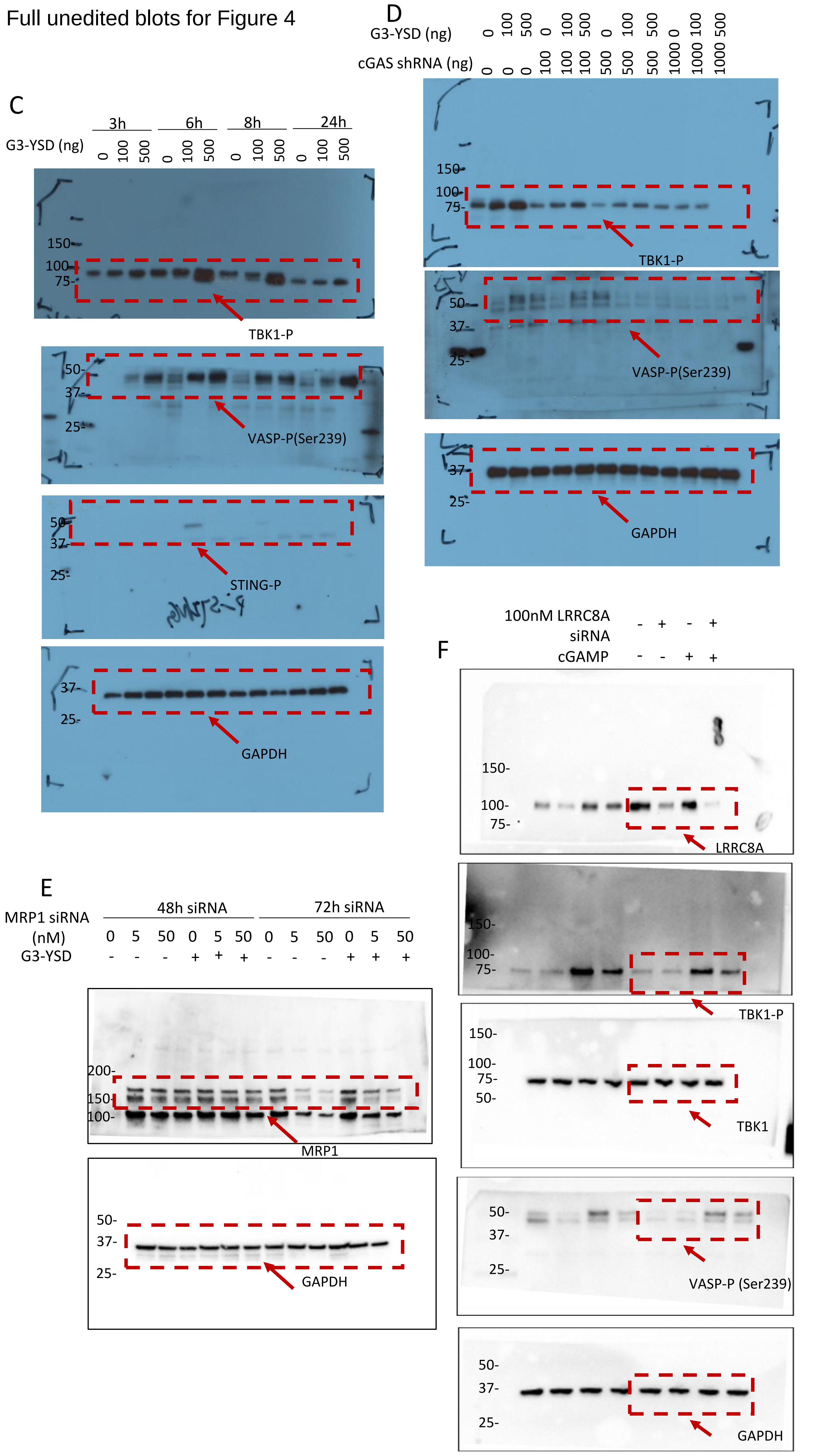

Supplement: Supplementary file 2 [file cir-148-1023-s002.pdf]
